# Open Access versus Subscription Access Journals of Paediatric Surgery

**Published:** 2015-01-01

**Authors:** Jamshed Akhtar, Bilal Mirza

**Affiliations:** 1 Department of Pediatric Surgery, National Institute of Child Health, Karachi; 2 Department of Pediatric Surgery, The Children’s Hospital and the Institute of Child Health, Lahore

Open access journals provide an opportunity to read quality research articles through internet across the world. Concern has been shown as to the standard of articles published in open access journals and their peer review process but same has been refuted by a study of Björk and Solomon.[1] They have concluded that scientific impact of open access journal is now approaching that of subscription journals.

In paediatric surgery, the impact of open access journals has not been evaluated presently but hopefully as they receive impact factor in future, this issue shall be addressed. There is encouraging trend towards addition of open access journals of paediatric surgery to international databases which in itself reflects quality of such publications. But unfortunately, most of the open access journals originate from developing countries. Few of these are “Electronic-only” publications. This takes care of cost of publication and therefore become economical and viable option on the longer run. The foremost issue is why journals from technologically advanced countries published by giants in the field of publications still charging huge amount both from the contributors as well as readers. The cost is huge not within the reach of researchers, students, residents etc from developing countries. This amounts to putting hurdles for researchers from developing world where the need of research is most. This gap is now being filled by open access journals and the number of manuscripts being sent to them and their quality has visibly improved. Same trend is apparent in our APSP Journal of Case Reports (AJCR). This is indexed with international databases and we hope to get impact factor soon. We have no conflict of interest as this is an official journal of The Association of Paediatric Surgeons of Pakistan (APSP). There are no subscription and publications charges thus researchers are encouraged to send their quality manuscripts for publication to this journal. Our peer review system further ensures that all submissions are seen by experts in the field to improve standard of the manuscript. We are thankful to all of our contributors and reviewers for their hard work.

## Footnotes

**Source of Support:** Nil

**Conflict of Interest:** None declared

